# The influence of geographic and climate factors on the timing of dengue epidemics in Perú, 1994-2008

**DOI:** 10.1186/1471-2334-11-164

**Published:** 2011-06-08

**Authors:** Gerardo Chowell, Bernard Cazelles, Hélène Broutin, Cesar V Munayco

**Affiliations:** 1 Mathematical and Computational Modeling Sciences Center, School of Human Evolution and Social Change, Arizona State University, Tempe, AZ, USA; 2 Division of Epidemiology and Population Studies, Fogarty International Center, National Institutes of Health, Bethesda, MD, USA; 3 UMR 7625, UPMC-CNRS-ENS, Ecole Normale Superieure, 46 rue d'Ulm, 75230, Paris cedex 05, France; 4 UMMISCO, UMI 209, IRD-UPMC, 32 avenue Henri Varagnat, 93142 Bondy cedex, France; 5 MIVEGEC, UMR CNRS 5290-IRD 224-UM1-UM2, 911 Avenue Agropolis, BP 64501, 34394 Montpellier Cédex 5, France; 6 Dirección General de Epidemiología, Ministry of Health. Calle Rivero de Ustariz 251. Jesús María-Lima 11, Perú

**Keywords:** Dengue, dynamics, community size, wavelet analysis, wavelet coherence, epidemic timing, climatic factors, Perú

## Abstract

**Background:**

Dengue fever is a mosquito-borne disease that affects between 50 and 100 million people each year. Increasing our understanding of the heterogeneous transmission patterns of dengue at different spatial scales could have considerable public health value by guiding intervention strategies.

**Methods:**

Based on the weekly number of dengue cases in Perú by province, we investigated the association between dengue incidence during the period 1994-2008 and demographic and climate factors across geographic regions of the country.

**Results:**

Our findings support the presence of significant differences in the timing of dengue epidemics between jungle and coastal regions, with differences significantly associated with the timing of the seasonal cycle of mean temperature.

**Conclusions:**

Dengue is highly persistent in jungle areas of Perú where epidemics peak most frequently around March when rainfall is abundant. Differences in the timing of dengue epidemics in jungle and coastal regions are significantly associated with the seasonal temperature cycle. Our results suggest that dengue is frequently imported into coastal regions through infective sparks from endemic jungle areas and/or cities of other neighboring endemic countries, where propitious environmental conditions promote year-round mosquito breeding sites. If jungle endemic areas are responsible for multiple dengue introductions into coastal areas, our findings suggest that curtailing the transmission of dengue in these most persistent areas could lead to significant reductions in dengue incidence in coastal areas where dengue incidence typically reaches low levels during the dry season.

## Background

Dengue fever is the most prevalent vector borne disease in the Americas and the second most prevalent in the world after malaria with 50-100 million annual dengue infections in tropical and subtropical regions [1]. Dengue is caused by four serotypes (DENV-1, DENV-2, DENV-3, and DENV-4) of the genus *Flavivirus *[1] and is transmitted by the mosquito species *Aedes aegypti *and *Aedes albopictus*. The severity of dengue disease ranges from asymptomatic, clinically non-specific with flu-like symptoms, dengue fever (DF), dengue hemorrhagic fever (DHF), and dengue shock syndrome (DSS) [1,2].

In Perú, dengue serotype DENV-1 was identified in 1990 [3], and it was followed by the invasion of American genotype DENV-2, responsible for the large dengue epidemic of 1995-1996 [4]. All four dengue serotypes co-circulated in Perú during the large epidemic of 2000-2001 (Asian DENV-2, DENV-3, and DENV-4 identified for the first time) [5] and were reported present in other countries of the region by 2000 [6].

Environmental factors (e.g., climatological, geographic) [7] and population mobility at different temporal and spatial scales [8] have been found to affect the spatio-temporal patterns of dengue transmission. For example, climatic conditions not only affect the development, maturation and survival of the vector *Ae. aegypti *[9-11] but also its role in dengue transmission. Specifically, the time for infected female mosquitoes to become infectious after biting an infectious individual (known as the extrinsic incubation period) is influenced by ambient temperature [12-14]. Moreover, the main dengue vector *Ae. aegypti *typically travels short distances [15], but human mobility patterns have been shown to contribute significantly to the heterogeneous transmission patterns of dengue at different spatial scales (community, provincial, regional) [8].

Achieving dengue eradication is a complex and challenging task. Mosquito eradication programs targeting *Ae. aegypti *in the Americas began in the 1950s and led to dramatic reductions in dengue incidence [16]. However, these programs were interrupted in the 1970s throughout the Americas facilitating dengue re-emergence in various regions of Central and South America with *Ae. aegypti *as the primary vector [16].

To date, there is no clinically efficient diagnostic test, drugs or vaccines available to date to fight dengue, and the epidemiology of the disease is still poorly understood [17,18]. Given these limitations, improving our understanding of the complex transmission dynamics of dengue at different spatial scales may be useful in the development and evaluation of alternative control measures [16,19-22]. For instance, the strategic allocation of limited resources for vector control programs could lead to improved control outcomes [23,24].

In this study we use spatial data of dengue incidence stratified by province and region as well as climate and demographic variables to study the transmission dynamics of dengue across the jungle, coast and mountain regions in Perú. In particular, dengue persists in the jungle region of Peru all year round and dengue outbreaks peak most frequently near March when rainfall is abundant. We explore the possibility that highly persistent jungle regions and cities of other dengue-endemic neighboring countries may be the source of outbreaks in coastal regions which are more likely to occur and become a public health concern during April-May right after the summer rain season.

## Methods

**Figure 1:**
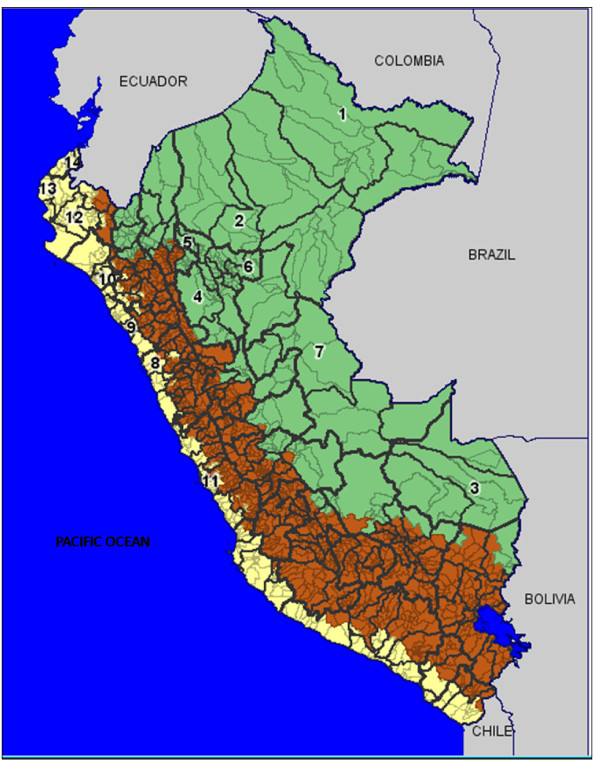
**Map of Peru with provincial divisions for 195 provinces and meteorological stations**. The geography of Peru covers a range of features, from a western coastal plain (yellow), the Andes Mountains in the center (brown), and the eastern jungle of the Amazon (green). The numbers indicate the local meteorological stations for 14 provinces that were representative of jungle (1-7) and coastal (8-14) regions.

Perú is a South American country located on the Pacific coast between the latitudes: -3 degrees S to -18 degrees S, sharing borders with Bolivia, Brazil, Chile, Colombia, and Ecuador (Figure 1). The population size is about 28 million heterogeneously distributed individuals over 1,285,220 km^2^, including a western coastal plain, the eastern jungle of the Amazon and the Andes Mountains, which separates the coastal from the jungle region (Figure 1). Politically Perú is divided into 25 administrative regions composed of 195 provinces ranging in population size from 7000 to more than 7 million people [25].

Perú's weather varies from tropical to temperate. The jungle (rainforest) is dry but hot during the dry season (May-October) and wet and hot, from November to April with temperatures reaching 36°C and heavy rainfall that causes river flooding on the smaller tributaries [26]. The mountain range region also has a May-October dry season, but is characterized by clear, sunny days and cold nights. November-April is the rainy season, with heaviest rainfall during the months of January and February and a mild daytime temperature that drops at night. The coastal region has an April-November winter season, with cloudy and cool days, and a hot and dry summer during December-March. The northern coast typically has higher temperatures and rainfall in the summer.

### Epidemiological Data

We obtained the weekly number of dengue cases at the province level during the period 1994-2008 from the Health Ministry's Dirección General de Epidemiología that is in charge of Perú's passive epidemiological surveillance system comprised of a network of over 7000 geographically distributed notifying units that includes 95% of the health centers. The surveillance network has collected weekly disease data reports since 1994 [27]. The case definitions for probable and confirmed dengue cases in use are those specified by the World Health Organization (WHO) guidelines [28] namely a dengue case was classified as probable whenever fever or chills were present in addition to at least two symptoms among myalgia, arthralgia, retro-orbtial pain, headache, rash, or some hemorrhagic manifestation (e.g., petechiae, hematuria, hematemesis, melena). Seventy-nine out of one hundred and ninety-five provinces reported dengue cases sometime during the period of interest (1994-2008). Only eighteen percent of the probable dengue cases were confirmed via anti-dengue IgM antibody tests by the regional laboratories under the supervision of Perú's National Institute of Health.

### Population, geographic, and climate data

Annual population size estimates of the Peruvian provinces during the years 1994-2008 were obtained from the National Institute of Statistics and Informatics [29]. Each province is classified according to its geographic location as coastal, mountain, or jungle area (Figure 1). Summary statistics for the population size and density and altitude of the 79 provinces examined in this study are given in Table 1. The population density of each province (people/km^2^) was estimated by dividing the province population size by the surface area (km^2^)[30]. The latitude and longitude coordinates of each province were also available [31]. Time series of the mean, minimum and maximum temperature and precipitation for the period of interest were obtained for 14 provinces according to existing local meteorological station coverage. But these data were representative of Coastal and Jungle regions (see Figure 1). We provide descriptive statistics of the climatic variables in jungle and coastal regions in Table 2.

**Table 1:** Summary statistics (median and range) of population size, population density (people/km^2^) and altitude of the provinces reporting dengue sometime during the study period (1994-2008) stratified by geographic region

Geographic region	Num. of Provinces	Population (median, range)	Population Density (median, range)	Altitude (median, range)
**Jungle**	28	64317 (7662, 503095)	7.9 (0.4, 38.0)	304 (124, 1835)

**Mountain**	25	62105 (18470, 285770)	20.7 (3.52, 91.4)	2294 (184, 4864)

**Coast**	26	130220 (15648, 6723130)	47.9 (7.4 - 5264)	192.5 (1, 4238)

**Table 2:** Summary statistics (mean and range) of the climate time series (1994-2008) using available province level data from jungle and coastal regions

Geographic region	Num. of Provinces	Mean Temp. (mean, range)	Min. Temp. (mean, range)	Max. Temp. (mean, range)	Precipitation
**Jungle**	7	26.6 (25.0, 27.7)	21.0 (19.0, 22.4)	31.9 (30.1, 33.5)	0.1 (0.03, 0.3)

**Coast**	7	22.1 (19.6, 25.8)	18.6 (16.3, 22.0)	26.8 (23.6, 30.8)	0.013 (0, 0.11)

### Timing of dengue epidemics and persistence patterns

A dengue epidemic was defined conservatively as the occurrence of 5 or more dengue cases during three or more consecutive weeks in a province, as used in a previous study [16]. This conservative definition was used to limit the confusion that results from imported cases (e.g., cases generated by humans visiting other provinces). Furthermore, cases considered to be part of an epidemic were recorded in a window of time bounded above and below by the absence of dengue cases for at least two consecutive weeks. We identified 380 dengue outbreaks in 1994-2008 as defined by this conservative definition. Furthermore, the total number of identified outbreaks was insensitive to the size of the threshold outbreak size. For each dengue epidemic, we computed the epidemic size (total number of dengue cases occurring during the epidemic) and the epidemic peak timing (week of the year at which the epidemic peak occurs).

We also explored the presence of a 'critical community size' [32] at the province level and across geographic regions above which dengue epidemics would typically take off. For this purpose, during the period of study, we quantified the relationship between population size and the proportion of weeks with dengue reports [16].

We scaled the dengue epidemics by their peak size in order to analyze the distribution of the timing of dengue epidemics throughout the years and across geographic regions. We studied the annual profiles of the average timing of dengue epidemics in jungle, coastal and mountain regions as well as the variability of the epidemic peak timing at the province level throughout the year during the study period.

To partially account for the introduction of new serotypes in Perú, two time periods were assessed in our analysis: 1994-1999 (1^st ^time period) and 2000-2008 (2^nd ^time period). These time periods correspond to the time intervals before and after all four dengue virus serotypes started to co-circulate in Perú [6].

### Association between Dengue Activity and Variation in Temperature

We quantified the association between dengue activity and ambient temperature across geographic regions by correlating the mean temperature at week i with the number of dengue cases by date of symptoms onset at week i+τ where τ is the natural time lag (in weeks) for changes in temperature to be reflected on dengue activity. This natural time lag accounts for the development, maturation and survival of the vector *Ae. aegypti *[8-10] as well as the extrinsic incubation period in the vector and the intrinsic incubation period in the human host [1,3]. The average occurrence of dengue (N_T_) in different temperature domains (T to T+ΔT) by geographic region was calculated as follows [33]:

where i is an index for weeks during the study period from 0 to n, t_i _the mean temperature for week i, C(i) the total number of dengue cases at week i, and f(t) a function which

The numerator of the above ratio represents the sum of all dengue cases at week i+τ falling within the temperature domain T to T+ΔT at week i. The denominator is the total number of occasions with T < t_i _< T+ΔT during the entire period of study. We set ΔT = 0.5°C. Our results were not sensitive to varying the time lag τ in range 1-3 weeks.

### Wavelet time series analysis

Wavelet time series analysis can be used to disentangle the non-stationary temporal evolution of the periodic components in the incidence of infectious diseases and periodic components of phenomena in other ecological systems (e.g., [19,21,34]). Here we use wavelet analysis (wavelet power spectrum, oscillations in a given periodic band, wavelet coherency) to investigate variations in the dominant periodic cycles across the time series of dengue by geographic region, quantify the non-stationary relationship between dengue time series and climate variables through wavelet coherence analysis and compute the instantaneous time lag between two time series (Additional file 1). These wavelet analyses have been performed using the Morlet wavelet as in previous studies [21,34,35]. Weekly time series were square root transformed to manage the variability in the amplitude of the time series [21,35,36] and statistically significance levels were determined using a 'beta surrogate' test as described in [37]. We performed the wavelet time series analyses using well-established algorithms implemented in Matlab (The Mathworks, Inc.)[21].

## Results

### Characteristics of dengue epidemics

**Figure 2:**
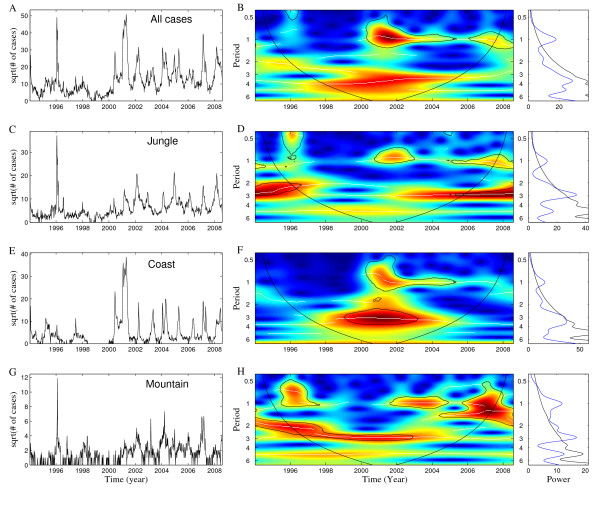
**Dengue incidence and wavelet power spectrum across geographic regions**. The weekly number of dengue cases by date of symptoms onset reported through the surveillance system in Perú during the period 1994-2008 (square root transformed) in A) the entire country, C) jungle region, E) coast region, and G) mountain region and the corresponding wavelet power spectrum of the weekly series of dengue fever for B) the entire country, D) jungle region, F) coast region, and H) mountain region. The color code for power values ranges from dark blue, low values, to dark red, high values. Statistically significant areas (threshold of 5% confidence interval) in wavelet power spectrum (left panels) are highlighted with solid line; the cone of influence (region not influenced by edge effects) is also indicated. Finally, the right panels show the mean spectrum (solid blue line) with its significant threshold value of 5% (solid black line).

A total of 86,631 dengue cases were reported during the 15-year study period. The aggregated epidemic curve shows a high degree of non-stationarity (Figure 2A). Wavelet time series analysis indicated a strong annual component around the time when all four dengue virus serotypes started to co-circulate in Perú (2000-2001) and a significant 3-4 y periodic component during 1998-2004 (Figure 2B).

Most of the dengue cases during the study period were reported in the jungle and coastal regions (47% and 49%, respectively) while only 4% of the dengue cases were reported in the mountain region. The amplitude of dengue epidemics at the province level in Perú is highly irregular across jungle, mountain and coastal regions (Additional file 2, Figures S1, S2 and S3). Epidemics in jungle regions often peak before those in the coastal regions by a few weeks and most notably after the large epidemics of 2000-2001 when all four dengue virus serotypes started to co-circulate in Perú (Figure 2C-H).

Wavelet time series analysis by geographic region revealed significant periodicity of 1-yr and 2-3 yr components (Figure 2D, F, H), which is in broad agreement with the periodicity range of the dynamics of dengue hemorrhagic fever in Thailand [19,21]. Moreover, the time span over which these patterns were statistically significant varied across geographic regions. The jungle region is dominated by a significant 1 yr component after 2000, the mountain province shows a significant 2-3 yr component before 2003 and a significant 1 yr component after 2003, and the Coastal region is characterized by both a significant 1 yr and a 3 yr components but just for the time period 2000-2003 (Figure 2D, F, H). Interestingly, a highly significant 0.5-1 yr component exists for each large epidemic associated with single or multiple serotype introductions, in 1996 for jungle and mountain regions and in 2001 for the coastal region (Figure 2D, F, H).

### Persistence patterns

Dengue is most persistent in jungle populations with dengue reports for 94.23% of the weeks of the study period (1994-2008). Coastal and mountain regions report dengue cases for 78% and 69% of the weeks of the study period (1994-2008), respectively. At the finer scale of provinces in jungle regions, we found the provinces of Alto Amazonas and Atalaya had the highest dengue persistence levels, with dengue reports for 82.7% and 64.5% of the weeks of the study period (1994-2008), respectively.

Dengue persistence in jungle provinces was positively correlated with population size (Spearman rho = -0.75, P < 0.0001, Additional file 2, Figure S4), suggesting the presence of a critical community size. Specifically, less than 30% of the weekly records had zero dengue incidences whenever the population was greater than 500,000 people [16].

### Temporal distribution of epidemics

**Figure 3:**
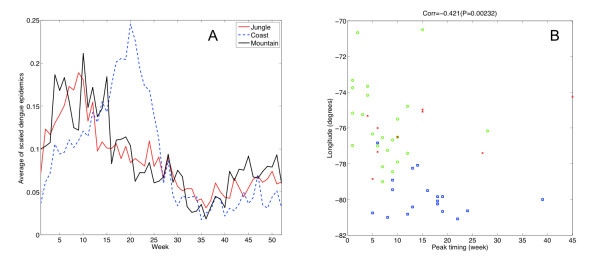
**Timing and persistence of dengue epidemics across geographic regions in Peru**. A) The average annual timing profile of dengue outbreaks in jungle, mountain and coastal regions obtained from the average of the scaled dengue epidemics at the province level; B) Epidemic peak timing as a function of the longitude coordinates of provinces across jungle (green circles), mountain (red crosses) and coastal (blue squares) regions in Perú. The peak timing of dengue epidemics is significantly correlated with the longitude coordinate of the provinces (Spearman rho = -0.42, P = 0.0023).

**Figure 4:**
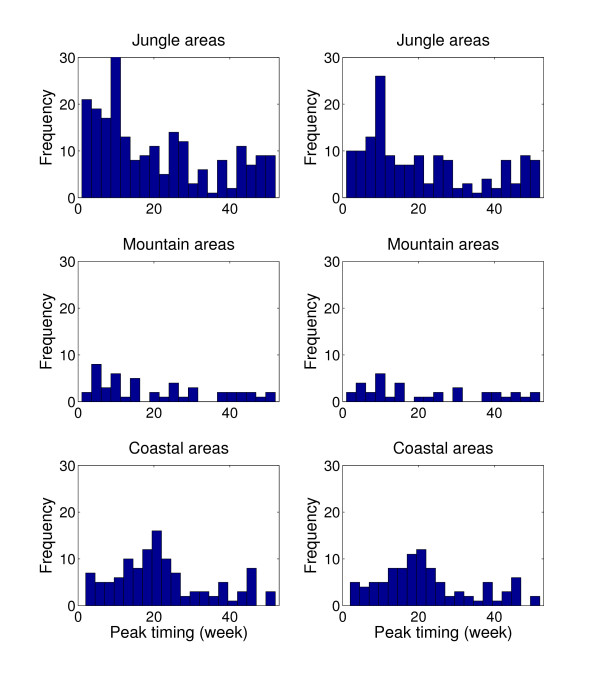
**Timing of dengue epidemics across geographic regions**. Left panels show the distribution of the peak timing of dengue outbreaks during the entire study period (1994-2008) and right panels show the distribution of the peak timing of dengue outbreaks occurring after 2000 when all four dengue virus serotypes started to co-circulate in Peru.

The annual timing profile of dengue across geographic regions showed that epidemics in the jungle and mountain regions peak prior to those in the coastal regions by about 6 weeks on average (Figure 3A, Additional file 2, Figure S5), with significant variablility of the epidemic peak timing throughout the year and across provinces during the entire study period (Figures 4) or during 2000-2008, when all four dengue serotypes started to co-circulate in Perú (Figure 4). Most epidemics peaked earlier in the jungle and a few weeks later in the coastal provinces. This finding was reinforced by studying the relationship between the average epidemic peak timing across provinces as one "moves" from jungle provinces to mountain and coastal provinces (see map in Figure 1). For this purpose, we used the longitude coordinate of each of the relevant provinces as a spatial proxy. We found the average timing of epidemic peaks to be moderately correlated with the corresponding longitude coordinate of the provinces (Spearman rho = -0.42, P = 0.0023, Figure 3B. This relationship was only statistically significant during the study period when all four dengue serotypes started to co-circulate in Perú (2000-2008), but it was not significant during the first 6 years of the study period (1994-1999, Additional file 2, Figure S6). Our findings indicated that dengue epidemics in Perú peak most frequently around March in jungle provinces while epidemics peak most frequently during April-May in coastal provinces (Additional file 2, Figure S7).

### Periodicity and synchronization of dengue incidence across geographic regions

**Figure 5:**
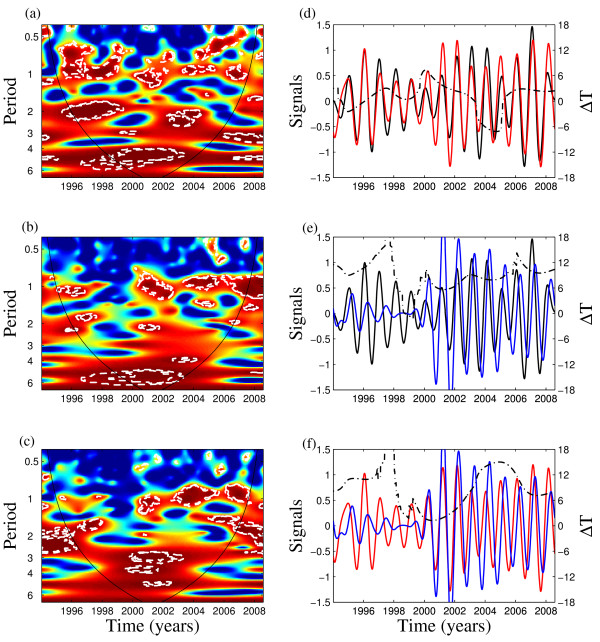
**The synchronization of dengue incidence across geographic regions of Perú**. Left panels show the wavelet coherence computed using the Morlet wavelet function from the weekly dengue incidence between jungle and mountain regions (a), mountain and coastal regions (b) and jungle and coastal regions (c), respectively. The colours are coded a dark blue, low coherence and dark red, high coherence. The dotted-dashed lines show 5% significance levels computed from 1000 bootstrapped series. The cone of influence indicates the region not influenced by edge effects. Right panels show the corresponding oscillating components computed with the wavelet transform in the 0.8-1.2 yr band for (d) jungle (red line) and mountain regions (black line), (e) mountain and coastal regions (blue line), and (f) jungle and coastal regions. The dot-dashed line is the instantaneous time delay in weeks (ΔT) between the oscillating components of two time series. (See [21] for the computation of this delay).

The analysis of wavelet coherence between the epidemics in the 3 regions revealed that the main significant coherence is for seasonal components (Figures 5A-5C). Next, we computed the oscillating components of the time series of the weekly dengue incidence in the 3 regions for the 0.8-1.2 yr band and also the instantaneous delay in the timing between these oscillating components in the 3 regions (Figures 5D-5F). Specifically this analysis shows for the seasonal component, the instantaneous time between the oscillating components in jungle and mountain regions varied with no delay on average (amplitude between plus or minus 6 weeks) underlying the synchronicity of the dengue epidemics in these regions (Figure 5D). Interestingly, our results also show that this instantaneous time between the oscillating components in jungle and coastal regions varied between 3 and 12 weeks within the period of high coherence that started near year 2000 (Figure 5F).

### Association of timing of epidemics with climate factors

**Figure 6:**
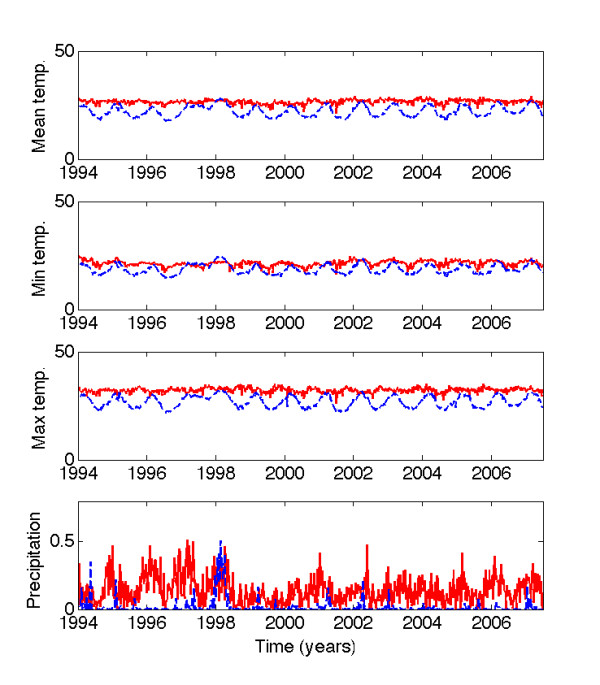
**Temperature and precipitation time series for jungle and coastal regions of Peru**. The average weekly time series of the mean, minimum and maximum temperature (°C) and precipitation (in) in jungle (red solid line) and coastal (blue dashed line) regions of Perú.

We hypothesized that the differences in the timing of dengue epidemics between jungle and coastal regions could be significantly associated with local climate (Figure 6). The wavelet coherence analysis of mean temperature and precipitation between jungle and coastal regions revealed that the main significant coherence occurred for the seasonal component for mean temperature, but the coherence pattern for the precipitation time series was less clear (Figures S8A-S8C).

**Figure 7:**
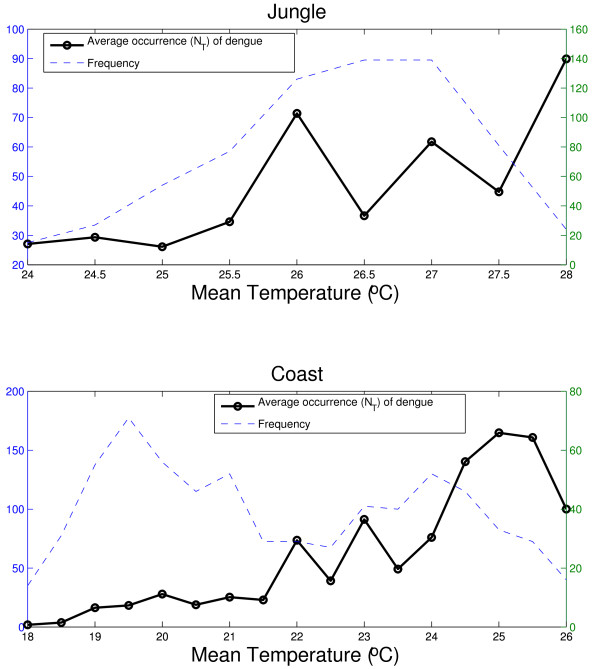
**Association between dengue activity and variation in temperature**. Average occurrence of dengue (NT) was defined as the average number of dengue cases observed for a given temperature domain (-o). Temperature ranges that occurred less than once per year during the study period are not included. The dashed line is given as a reference of the number of weeks with a given temperature domain during the entire study period using ΔT = 0.5°C and τ = 2 weeks.

**Figure 8:**
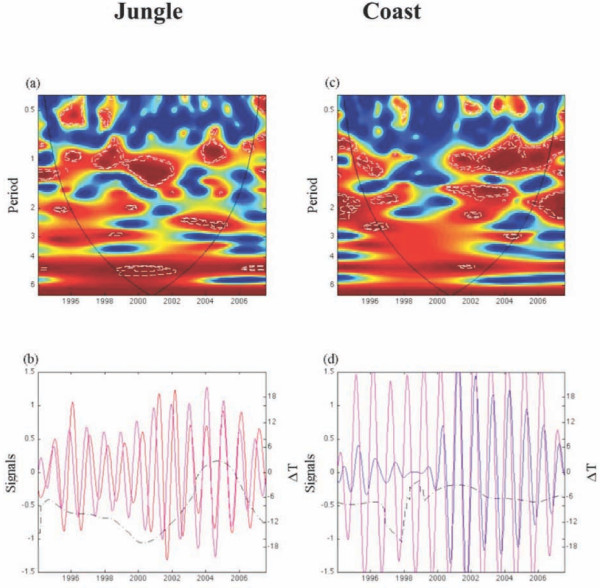
**The synchronization between dengue incidence and mean temperature between jungle and coastal regions of Perú**. Top panels show the wavelet coherence computed using the Morlet wavelet function from the weekly time series of dengue and mean temperature in (a) jungle and (c) coastal regions, respectively. The colors are coded as dark blue, low coherence and dark red, high coherence. The dotted-dashed lines show 5% significance levels computed from 1000 bootstrapped series. The cone of influence indicates the region not influenced by edge effects. Bottom panels show the corresponding oscillating components computed with the wavelet transform in the 0.8-1.2 yr band for (b) jungle (red line) and (d) coastal regions (blue line). The dot-dashed line is the instantaneous time delay in weeks (ΔT) between the oscillating components of two time series. (See [21] for the computation of this delay).

The instantaneous delay of the oscillating components of the time series of the weekly mean temperature for jungle and coastal regions in the 0.8-1.2 yr band varied consistently between 7-13 weeks (Additional file 2, Figure S8B). For precipitation the instantaneous time delay of the time series fluctuated over the study period from 0 to 6 weeks (Additional file 2, Figure S8D). Next, we analyzed the correlation and the coherence patterns between dengue incidence and mean temperature and precipitation. The average occurrence of dengue as a function of mean temperature domains (N_T_) revealed a strong association of these variables in both jungle (Spearman rho = 0.83, P = 0.005; Figure 7A) and coastal regions (Spearman rho = 0.95, P < 0.0002; Figure 7B). The coherence analysis indicated that the main significant coherence was for the seasonal component associated with mean temperature (Figures 8A and 8C) while this relationship was less clear with precipitation (Additional file 2, Figures S9A, S9B, S9C and S9D).

Overall, our findings support the hypothesis that there are significant differences in the timing of dengue epidemics between jungle and coastal areas, and these differences are associated with the timing of the seasonal cycle of mean temperature. Our results could also suggest that dengue is frequently imported into coastal regions through infective sparks from endemic areas including the jungle region of Perú and/or cities of other neighboring endemic countries, where propitious environmental conditions promote the stability of year round vector breeding sites.

## Discussion

We have combined highly refined spatial and temporal dengue incidence data together with geographic, demographic and climate information with the aim of uncovering dengue transmission patterns across geographic regions in Perú. Our findings suggest that there is a significant relationship between the timing of dengue transmission in jungle and coastal regions of Perú and local ambient temperature that affect the survival and development of the vector.

In jungle regions where dengue is highly persistent large outbreaks occur most frequently around March during the heavy rain season and could be the source of dengue outbreaks in coastal areas following the summer rainfall when propitious environmental conditions promote vector development. It is also possible that such dengue introductions, particularly into the northern coastal region, originate from cities of other neighboring countries. Indeed, the several-week difference in the period of highest dengue activity between the jungle and coastal region could be attributed to several factors including: (1) the natural delay of development of the vector from larva to adult stages (7-14 days) [38], (2) the time to successful invasion of the dengue virus into coastal areas, and (3) the generation interval of dengue, which is composed of several factors including the mean extrinsic (mosquito) incubation period (5-15 days) [39], the mean intrinsic incubation period (4-7 days), the mean host infectious period (3-7 days) [39] and the mean adult mosquito lifespan (6-15 days) [40]. Future dengue research in the region should test the hypothesis that jungle areas of Peru act as a reservoir that sparks dengue in other areas of the country.

Our results could have considerable public health implications for dengue control. If the jungle areas are responsible for multiple dengue introductions into coastal areas of Perú, our findings suggest that curtailing the transmission of dengue in areas of high dengue persistence could lead to significant reductions in dengue incidence in coastal areas where dengue incidence typically reaches quite low levels during the dry season. This is associated with low densities of the *Aedes *mosquito population in addition to a longer mosquito incubation period caused by lower temperatures [38]. The use of bed nets, mosquito repellents and screens in houses could have a significant effect reducing the effective population size to a level below a critical community size at which sparks of infection typically lead to stochastic 'fade-out' due to higher demographic stochasticity in small populations [19,41-43]. This intervention could have a particularly significant effect in jungle regions in Perú where the critical community size has been previously estimated at ~500,000 people [16].

Cultural factors pose some of the most challenging obstacles for dengue control worldwide. In Perú, limited access to running water in the northern coastal region causes people to accumulate water in open containers and wells, which are potential breeding sites for the mosquito *Ae. aegypti*. In addition, it is not uncommon to find stagnant water in peri-domestic containers in these areas (e.g., flower vases, pots and discarded tires). Therefore, vector control programs focused on eliminating breeding sites and reducing vector densities, should motivate populations to keep wells and water containers closed and to reduce the number of unnecessary objects near houses. Because of limited resource availability, these interventions would be most effective if conducted within a few weeks prior to the rainy season.

There are several caveats in our study. First, we assumed that incidence of probable dengue cases was a reliable indicator to define the spatial heterogeneity of dengue across Peruvian regions as in previous studies (e.g.,[16,44]). Second, our analyses were conducted with the assumption that reporting and coding of dengue cases was homogeneous across Peruvian areas and over the course of the entire study period. Third, serotype-specific dengue data over time and by geographic region were not available to analyze the spatial and temporal dynamics of dengue between jungle and coastal areas. It is worth noting that longitudinal field studies that monitor populations for dengue viruses have been carried out in Peru, but these have been limited to specific geographic regions [18,45]. Fourth, although we have made use of the best climatological data available, data from meteorological stations may not be necessarily representative of the climate patterns in the larger provinces. The use of interpolated climate data with better meteorological station coverage at smaller spatial scales (e.g., district level) is recommended. Finally, we relied on the provincial divisions of Peru, which are not necessarily the most meaningful spatial units for dengue disease dynamics [16,46].

## Conclusions

Dengue is highly persistent in jungle areas of Perú where epidemics peak most frequently around March when rainfall is abundant. Differences in the timing of dengue epidemics in jungle and coastal regions are significantly associated with the seasonal temperature cycle. Our results suggest that dengue is frequently imported into coastal regions through infective sparks from endemic jungle areas and/or cities of other neighboring endemic countries, where propitious environmental conditions promote year-round mosquito breeding sites. If jungle endemic areas are responsible for multiple dengue introductions into coastal areas, our findings suggest that curtailing the transmission of dengue in these most persistent areas could lead to significant reductions in dengue incidence in coastal areas where dengue incidence typically reaches low levels during the dry season.

The spatial differences in the timing of epidemics highlighted in our study demonstrate the advantages of high-resolution spatio-temporal data to detect heterogeneous levels in the spatio-temporal dynamics of dengue epidemics. The presence of significant levels of spatio-temporal heterogeneity highlights the need to exercise caution in the analysis of aggregated time series data that comprise significant spatial heterogeneity such as in the case of Perú.

## Competing interests

The authors declare that they have no competing interests.

## Authors' contributions

GC and BC conceived the study and analyzed the data; GC wrote the first draft of the manuscript. BC, HB and CM participated in the interpretation of results and in the writing and editing of the manuscript. All authors read and approved the final manuscript.

## Pre-publication history

The pre-publication history for this paper can be accessed here:

http://www.biomedcentral.com/1471-2334/11/164/prepub

## Supplementary Material

Additional file 1 **Supplementary methods**. Wavelet time series analysis Click here for file

Additional file 2 **Supplementary information**. Supplementary figures Click here for file
