# Supplementary material for: The influence of geographic and climate factors on the timing of dengue epidemics in Perú, 1994-2008
Source: BMC Infect Dis. 2011 Jun 8;11:164. doi: 10.1186/1471-2334-11-164 (PMC3121613; doi:10.1186/1471-2334-11-164)
Supplement: Additional file 1 — Supplementary methods. Wavelet time series analysis [file 1471-2334-11-164-S1.DOC]

**Wavelet time series analysis**

Wavelet time series analysis can be used to disentangle the non-stationary temporal evolution of the periodic components in the incidence of infectious diseases and periodic components of phenomena in other ecological systems (e.g., [1-4]). Here we use wavelet analysis (wavelet power spectrum, oscillations in a given periodic band, wavelet coherency) to investigate variations in the dominant periodic cycles across the time series of dengue by geographic region, quantify the non-stationary relationship between dengue time series and climate variables through wavelet coherence analysis and compute the instantaneous time lag between two time series.


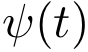
Wavelet analysis is based on the wavelet transform that decomposes signals over dilated and translated functions called “mother wavelets”, that can be expressed as the function of two parameters, one for the time position *τ*, and the other for the scale of the wavelets *a*:

where ‘ * ’ denotes the complex conjugate form and *x(t)* the signal.

As in most of the application of wavelets in Ecology and Epidemiology we employ the Morlet wavelets. Some discussion on the choice of the mother wavelet can be found in [1, 3-5].

An important point with the continuous wavelet is the relationship between the frequency and the wavelet scale that can be derived analytically [6]. For the Morlet wavelet, the relation between frequencies and wavelet scales is given by:

When , the wavelet scale *a* is inversely related to the frequency, . This greatly simplifies the interpretation of the wavelet analysis and one can replace, on all equations, the scale *a* by the period *1/f*.

In some sense, the wavelet transform can be regarded as a generalization of the Fourier transform and by analogy with spectral approaches one can compute the local wavelet power spectrum:

As given in Fourier analysis, the univariate wavelet analysis can be extended to quantify statistical relationships between two time series *x(t)* and *y(t)* by computing the wavelet coherency :

where <> indicates smoothing in both time and frequency; *W_x_( f, τ)* is the wavelet transform of series *x(t);* *W_y_( f, τ)* is the wavelet transform of series y(t); and is the cross wavelet power spectrum. The wavelet coherence provides local information about where two non-stationary signals, *x(t)* and *y(t)*, are linearly correlated at a particular frequency (or period). *R_x,y_( f, τ)* is equal to 1 when there is a perfect linear relationship at a particular time and frequency between the two signals [4].

For our analysis, weekly time series were square root transformed to manage the variability in the amplitude of the time series as in previous studies [1, 3, 7] and statistically significance levels were determined using a ‘beta surrogate’ test as described in [8] . We performed the wavelet time series analyses using well-established algorithms implemented in Matlab (The Mathworks, Inc.), which have been previously used to study dengue transmission patterns in Thailand [3] (http://www.biologie.ens.fr/~cazelles/bernard/Research.html).

**References**

1. Grenfell BT, Bjornstad ON, Kappey J: **Travelling waves and spatial hierarchies in measles epidemics**. *Nature* 2001, **414**(6865):716-723.

2. Cummings DA, Irizarry RA, Huang NE, Endy TP, Nisalak A, Ungchusak K, Burke DS: **Travelling waves in the occurrence of dengue haemorrhagic fever in Thailand**. *Nature* 2004, **427**(6972):344-347.

3. Cazelles B, Chavez M, McMichael AJ, Hales S: **Nonstationary influence of El Nino on the synchronous dengue epidemics in Thailand**. *PLoS Med* 2005, **2**(4):e106.

4. Cazelles B, Chavez M, Magny GC, Guegan JF, Hales S: **Time-dependent spectral analysis of epidemiological time-series with wavelets**. *J R Soc Interface* 2007, **4**(15):625-636.

5. Cazelles B, Chavez M, Berteaux D, Menard F, Vik JO, Jenouvrier S, Stenseth NC: **Wavelet analysis of ecological time series**. *Oecologia* 2008, **156**(2):287-304.

6. Meyers SD, Kelly BG, O'Brien JJ: **An Introduction to Wavelet Analysis in Oceanography and Meteorology: With Application to the Dispersion of Yanai Waves**. *Monthly Weather Review* 1993, **121**(10):2858.

7. Viboud C, Bjornstad ON, Smith DL, Simonsen L, Miller MA, Grenfell BT: **Synchrony, waves, and spatial hierarchies in the spread of influenza**. *Science* 2006, **312**(5772):447-451.

8. Rouyer T, Fromentin J-M, Stenseth NC, Cazelles B: **Analysing multiple time series and extending significance testing in wavelet analysis**. *Marine Ecology Progress Series* 2008, **359**:11-21.
