# Supplementary material for: The influence of geographic and climate factors on the timing of dengue epidemics in Perú, 1994-2008
Source: BMC Infect Dis. 2011 Jun 8;11:164. doi: 10.1186/1471-2334-11-164 (PMC3121613; doi:10.1186/1471-2334-11-164)
Supplement: Additional file 2 — Supplementary information. Supplementary figures [file 1471-2334-11-164-S2.DOC]

**Figure S1**

**Dengue incidence in jungle areas of Peru**.
Weekly dengue counts reported for the 15 provinces with the highest dengue burden in jungle regions of Peru during 1994-2008.


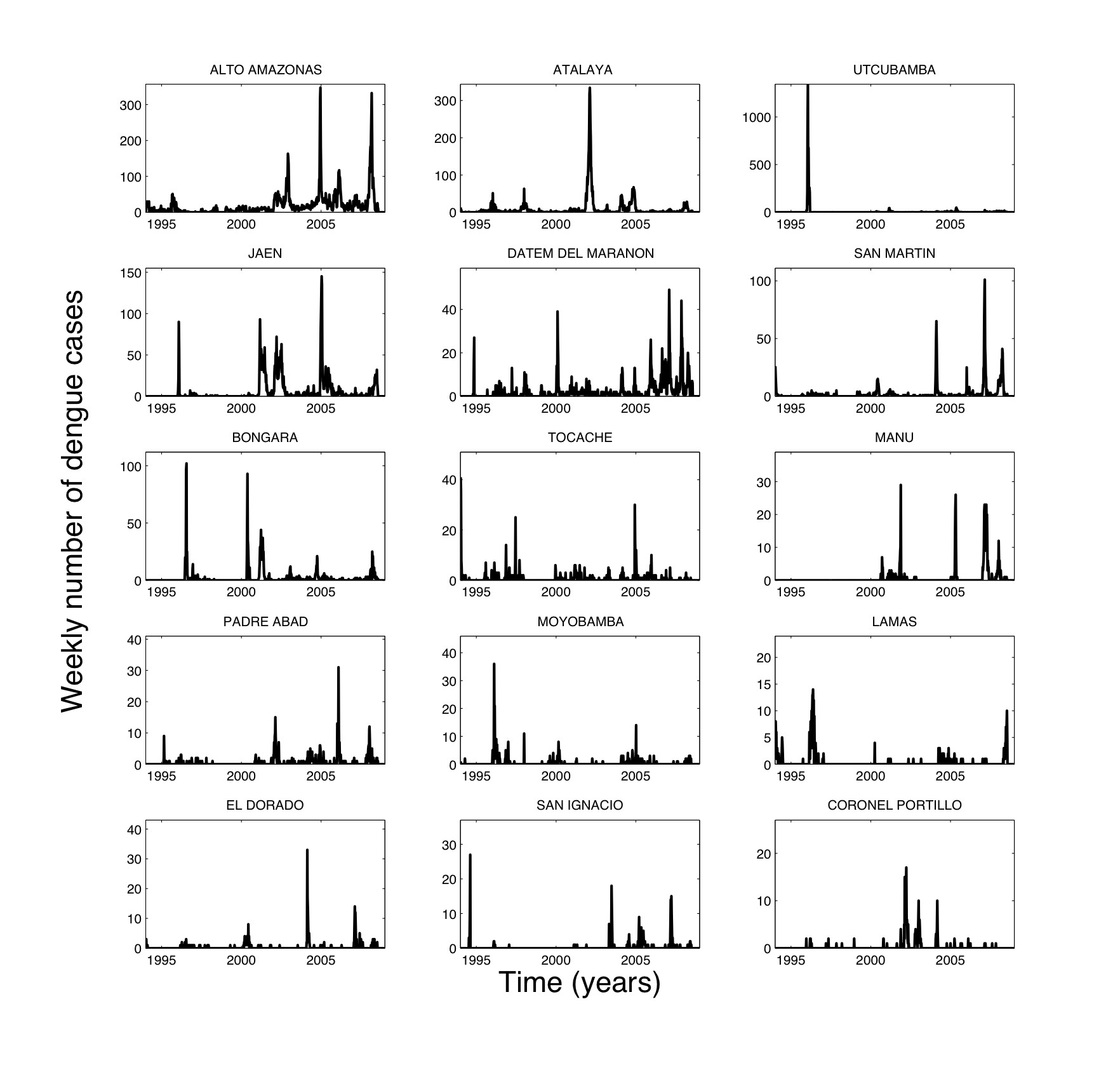


**Figure S2**

**Dengue incidence in mountain areas of Peru**.
The weekly dengue counts for the provinces with the highest dengue burden in mountain regions of Peru during 1994-2008.


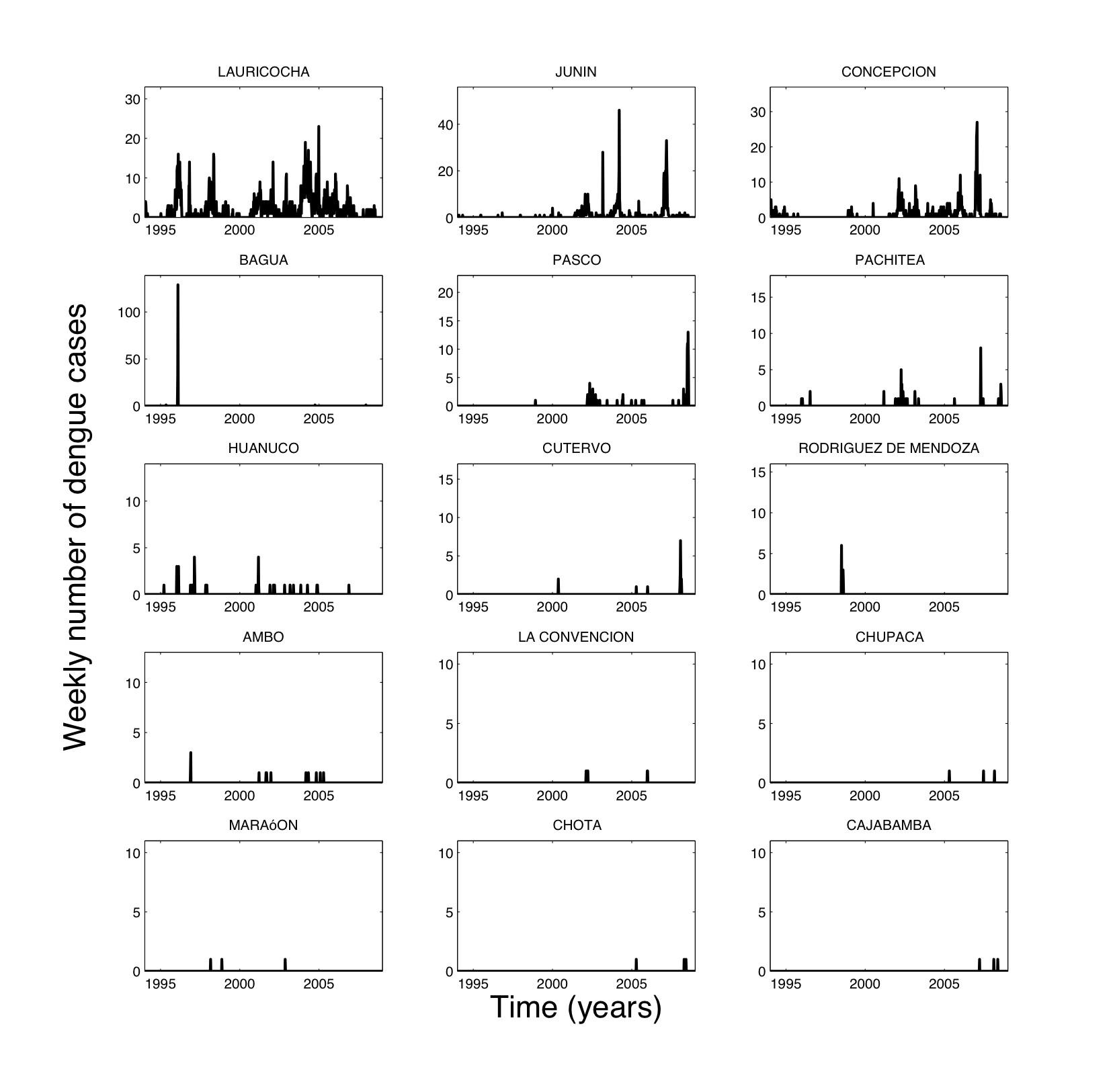


**Figure S3**

**Dengue incidence in coastal areas of Peru.**The weekly dengue counts for the provinces with the highest dengue burden in coastal regions of Peru during 1994-2008.


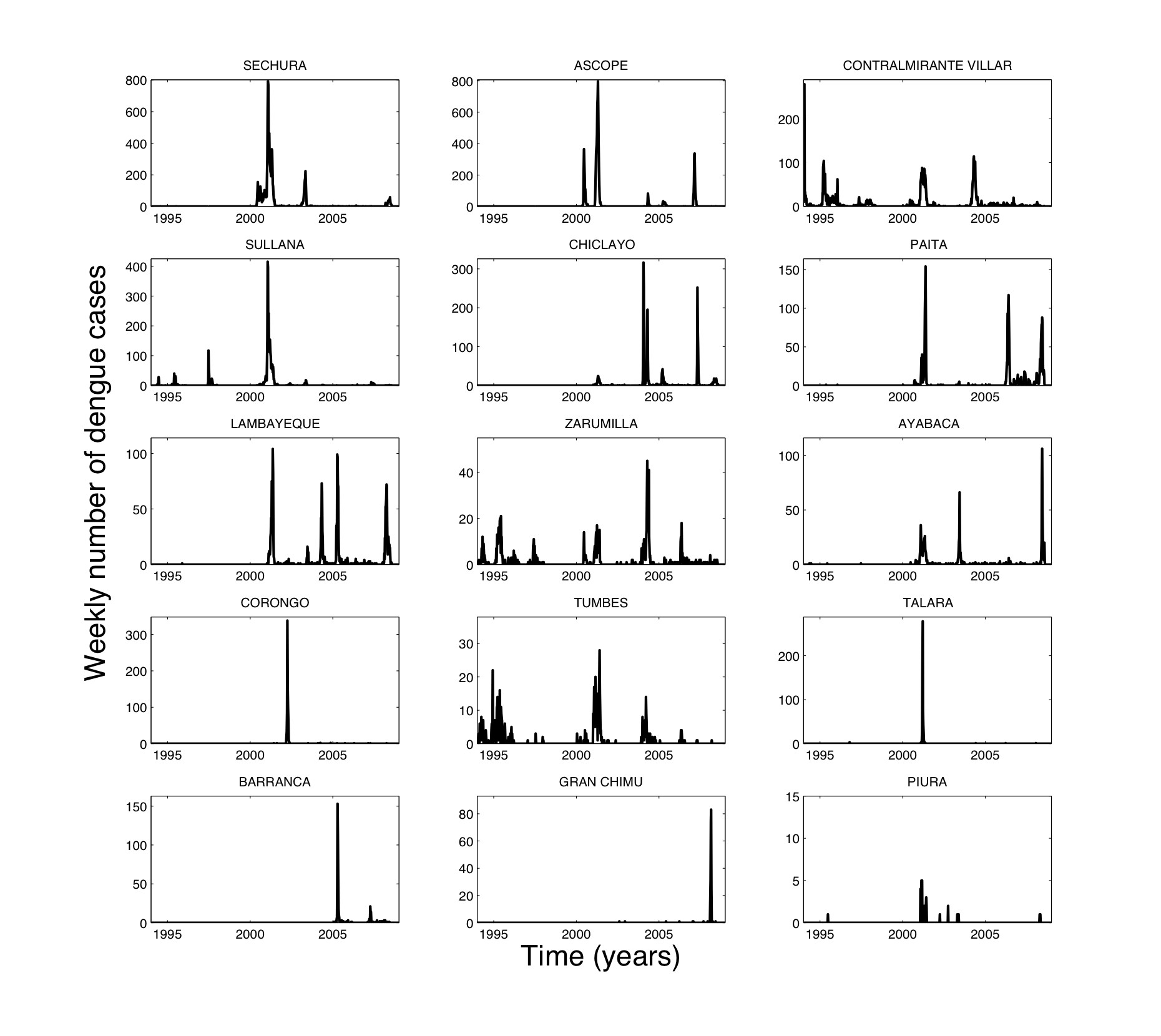


**Figure S4**

The proportion of weeks with dengue reports as a function of population size of provinces comprising the jungle region of Perú. The proportion of weeks with dengue reports (1994-2008) were positively correlated with population size in jungle regions (Spearman r=-0.75, P<0.0001) with >70% of weekly records with dengue reports in jungle regions with a population>500,000 people.

**
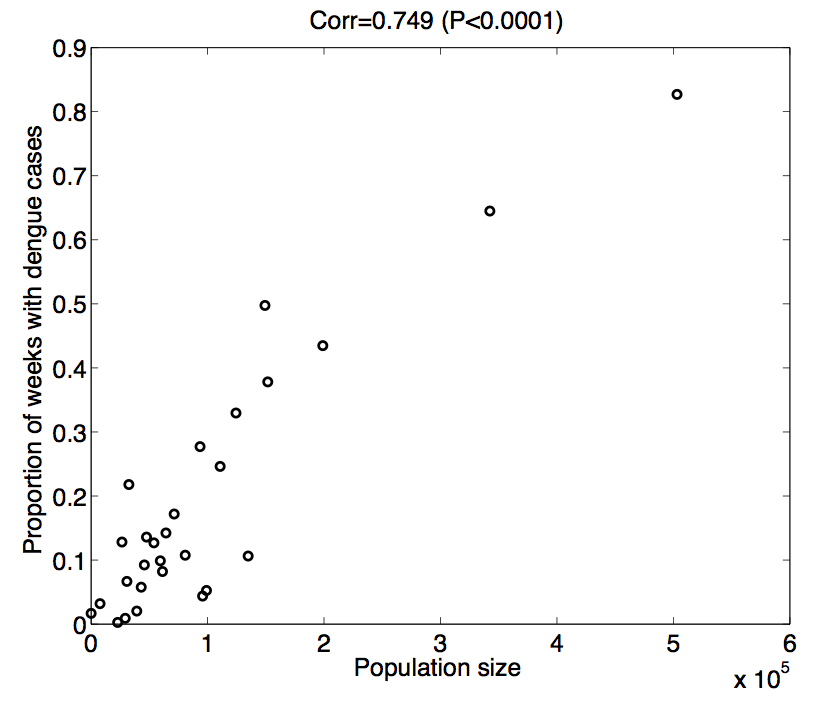
**

**Figure S5**. **Average annual timing profile of dengue incidence across geographic regions**.
The average annual timing profile of dengue incidence in jungle, mountain and coastal regions obtained from the average of the scaled dengue epidemics at the province level during the periods 1994-1999 (top) and 2000-2008 (bottom), prior and after all four dengue virus serotypes started to co-circulate in Peru.

| 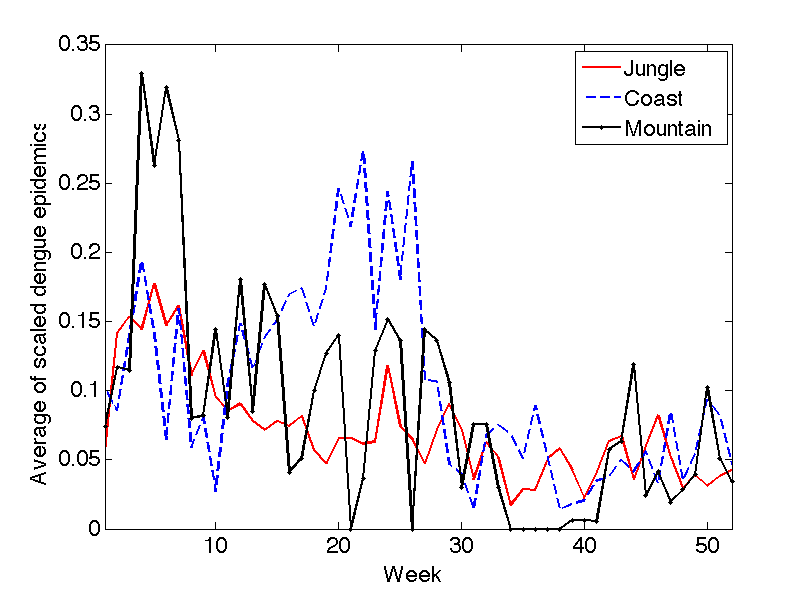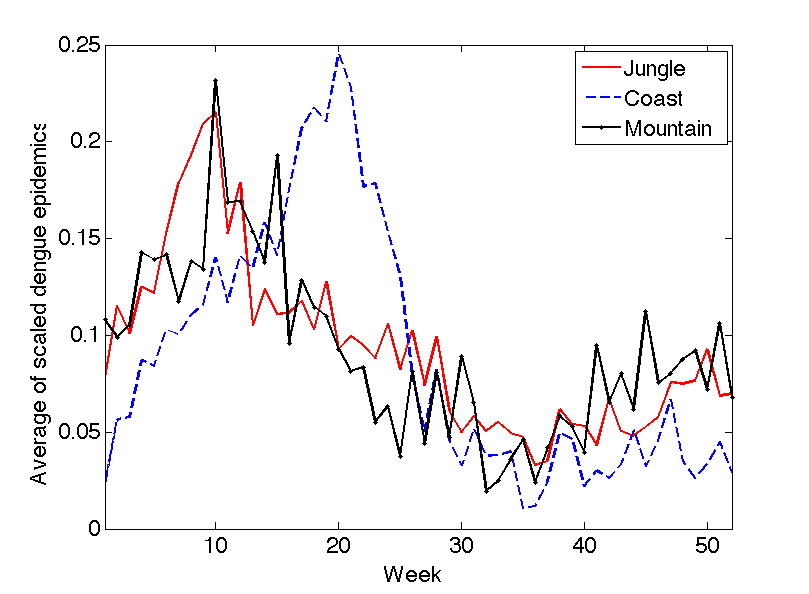  A  B |
| --- |

**Figure S6.**

**Average epidemic peak timing across provinces (1994-2008).**

Epidemic peak timing as a function of the longitude coordinates of provinces in Perú during the periods 1994-1999 (top) and 2000-2008 (bottom) prior and after all four dengue virus serotypes started to co-circulate in Peru. The peak timing of dengue epidemics taking place after 2000 is significantly correlated with the longitude coordinate of the provinces (Spearman rho=-0.34, P=0.02).

| 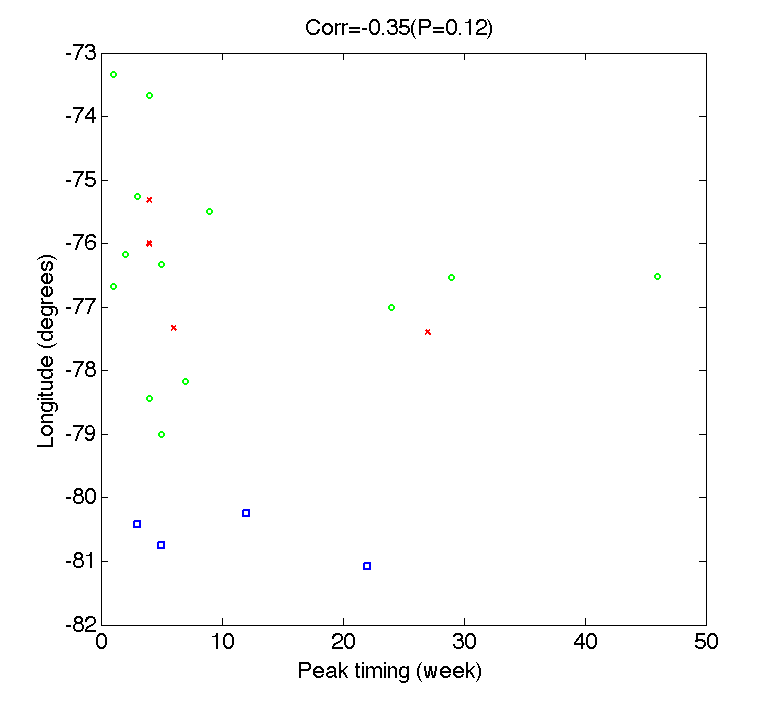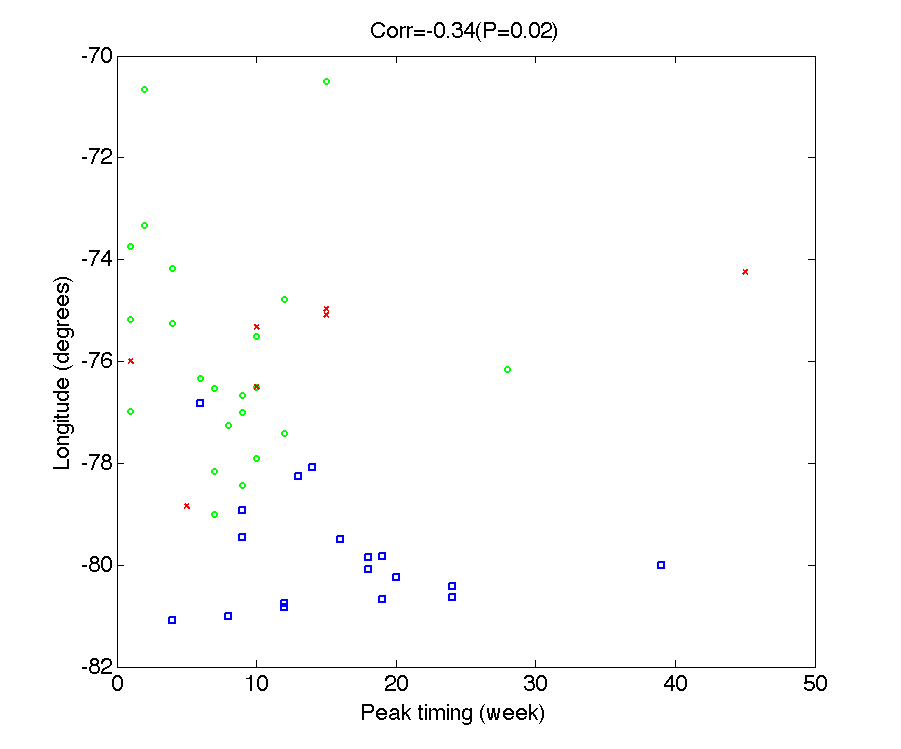  A  B |
| --- |

**Figure S7**

Map of Perú with provincial divisions differentiated with two different colors for provinces where epidemics peak most frequently before and during or after week 12 of the year. Provinces with no data are indicated in white of which the vast majority correspond to provinces located in at higher altitudes in the mountain region of Perú (see map in Figure 1).

**
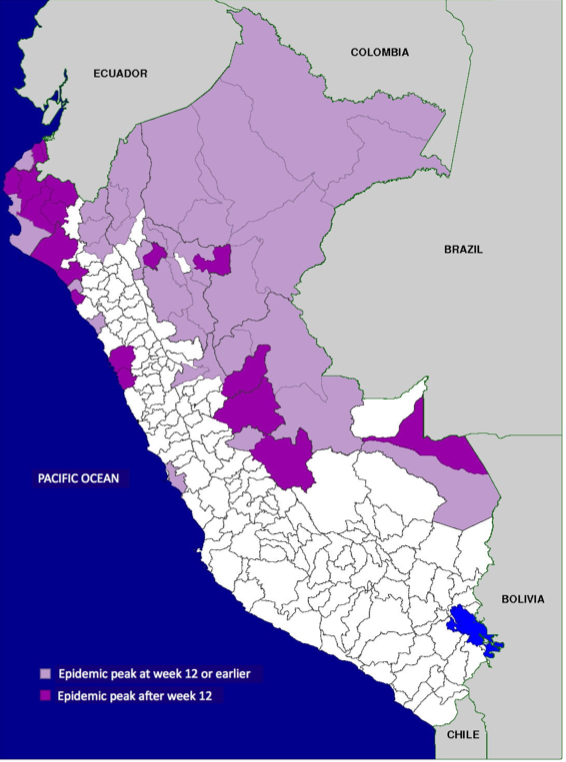
**

**Figure S8**

**The synchronization of mean temperature and precipitation between jungle and coastal regions of Perú.** Top panels show the wavelet coherence computed using the Morlet wavelet function from the average weekly time series of mean temperature (a) and precipitation (c) between jungle and coastal regions, respectively. The colors are coded as dark blue, low coherence and dark red, high coherence. The dotted-dashed lines show 5% significance levels computed from 1000 bootstrapped series. The cone of influence indicates the region not influenced by edge effects. Bottom panels show the corresponding oscillating components computed with the wavelet transform in the 0.8-1.2 yr band for (b) mean temperature in jungle (red line) and coastal regions (blue line) and (d) precipitation. The dot-dashed line is the instantaneous time delay in weeks (ΔT) between the oscillating components of two time series. (See [19] for the computation of this delay).

| **Mean Temperature** | **Precipitation** |
| --- | --- |
| 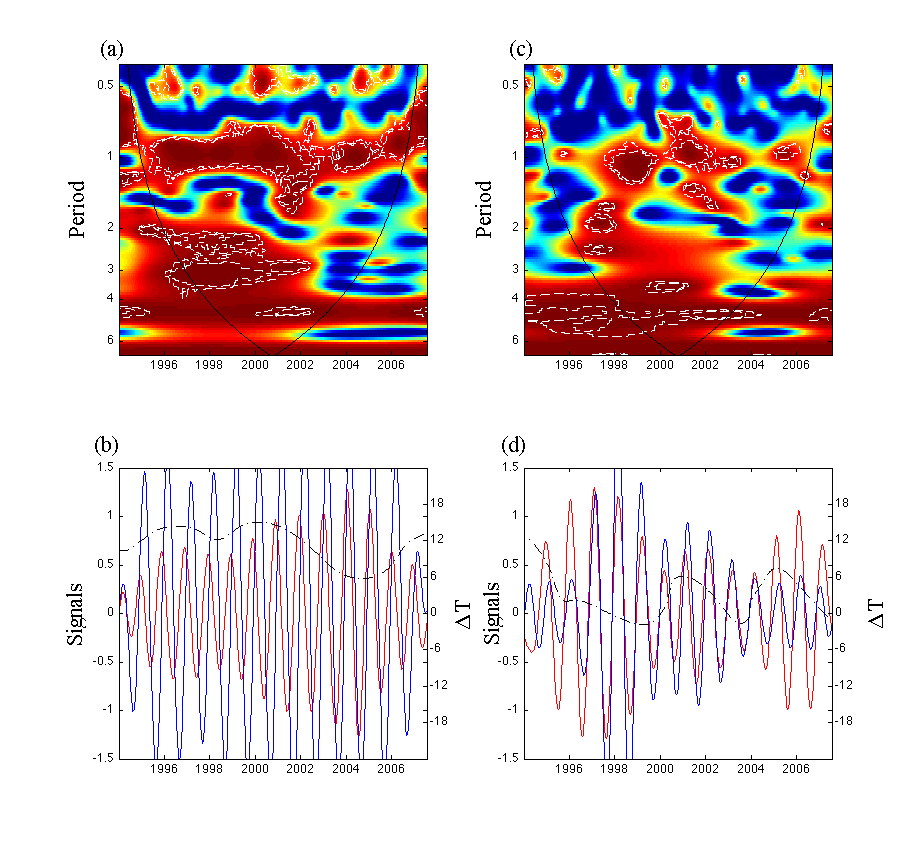 | |

**Figure S9**

**The synchronization between dengue incidence and precipitation between jungle and coastal regions of Peru.**

Top panels show the wavelet coherence computed using the Morlet wavelet function from the weekly time series of dengue and precipitation in (a) jungle and (c) coastal regions, respectively. The colors are coded as dark blue, low coherence and dark red, high coherence. The dotted-dashed lines show the 5% significance levels computed from1000 bootstrapped series. The cone of influence indicates the region not influenced by edge effects. Bottom panels show the corresponding oscillating components computed with the wavelet transform in the 0.8-1.2 yr band for (b) jungle (red line) (d) coastal regions (blue line). The dot-dashed line is the instantaneous time delay in weeks (ΔT) between the oscillating components of two time series. (See [13] for the computation of this delay).

| **Jungle** | **Coast** |
| --- | --- |
| 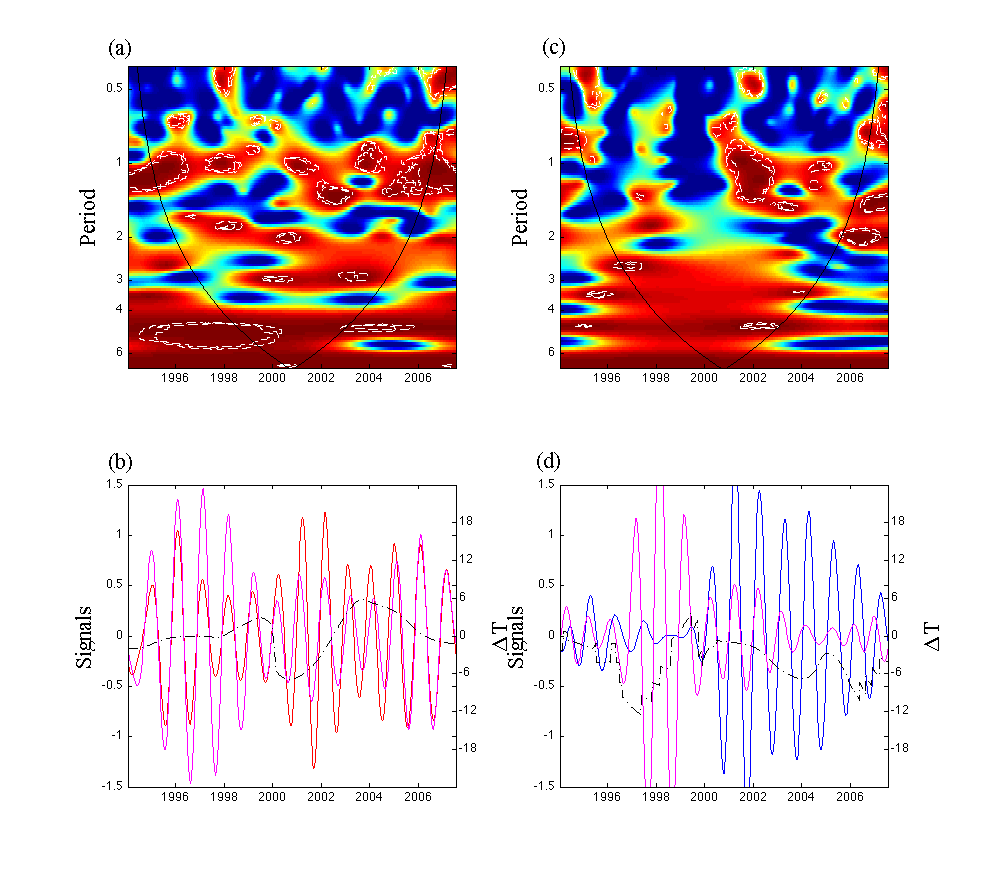 | |
